# Multisite musculoskeletal pain in the general population: a cross-sectional survey

**DOI:** 10.1590/1516-3180.2021.0134.R1.05052021

**Published:** 2021-11-08

**Authors:** Alberto de Vitta, Nicoly Machado Maciel, Thiago Paulo Frascareli Bento, Caio Vitor dos Santos Genebra, Sandra Fiorelli Almeida Penteado Simeão

**Affiliations:** I PT, PhD. Physiotherapist and Assistant Professor, Centro Universitário das Faculdades Integradas de Ourinhos, Ourinhos (SP), Brazil.; II PT, MSc. Doctoral Student, Faculdade de Medicina de Ribeirão Preto (FMRP), Universidade de São Paulo (USP), Ribeirão Preto (SP), Brazil.; III PT, MSc. Physiotherapist, Department of Physiotherapy, Universidade do Sagrado Coração (UNISAGRADO), Bauru (SP), Brazil.; IV PT, MSc. Physiotherapist, Department of Physiotherapy, Universidade do Sagrado Coração (UNISAGRADO), Bauru (SP), Brazil.; V PT, PhD. Assistant Professor, Department of Mathematics, Universidade do Sagrado Coração (UNISAGRADO), Bauru (SP), Brazil.

**Keywords:** Prevalence, Pain, Risk factors, Population characteristics, Epidemiology, Multisite musculoskeletal pain, Number of pain sites, Adult health

## Abstract

**BACKGROUND:**

Epidemiological studies focusing on multisite musculoskeletal pain have revealed that the prevalence of multisite pain is high in general populations.

**OBJECTIVE:**

To ascertain the prevalence of multisite musculoskeletal pain in the last 12 months and in the last seven days, in a population-based sample and investigate its association with demographic, socioeconomic, behavioral, reported morbidity and ergonomic variables.

**DESIGN AND SETTING:**

Cross-sectional population-based survey in Bauru, São Paulo (Brazil).

**METHODS:**

600 individuals were interviewed. The following data were collected: participants’ characteristics, through a precoded questionnaire; physical activity level, through the International Physical Activity Questionnaire; and musculoskeletal symptoms, through the Nordic questionnaire. Descriptive, bivariate and Poisson regression analyses were performed.

**RESULTS:**

The prevalence of multisite musculoskeletal pain was 46.5% (confidence interval, CI 42.5 to 50.5) in the last 12 months and 26.1% (CI 22.8 to 29.8) in the last seven days. The variables associated with multisite pain in the last 12 months were female sex, presence of hypertension, diabetes mellitus or depression, watching TV more than three times a week and working in a seated position. Formerly smoking was a protection factor. The variables associated with multisite pain in the last seven days were female sex, age group 60 years and over, low income, presence of comorbidities of hypertension, diabetes mellitus or depression and working in a seated position.

**CONCLUSION:**

There was high prevalence of multisite musculoskeletal pain, which was associated with demographic, socioeconomic, work-related, electronic device-related and reported morbidity variables.

## INTRODUCTION

Musculoskeletal pain is a significant burden for the community in several countries, and is one of the main health problems around the world. Over recent decades, many epidemiological studies have focused on multisite musculoskeletal pain, and their findings have revealed that the prevalence of multisite pain is high, both in the general and in the working population.^1,2^ The prevalence in the general population and among workers has been found to range from 2% to 60%,^1,3^ depending on the definitions of the pain site and study population.^4,5^

Epidemiological studies have found that multisite pain is associated with female sex, advanced age, educational level, obesity, smoking, depressive and/or anxiety disorders, low self-rated health^3,6^ and work-related factors: load handling, inadequate postures, repetitive movements, physical and mental stress, low support and job dissatisfaction.^4,7^

It has become important to obtain knowledge of the prevalence and factors associated with multisite pain in the general population, considering that, previously, a large number of studies focused on prevalence and risk factors in specific populations (workers) and single anatomical sites, such as the lumbar region.^8^ Multisite pain can interfere with the ability to work, functionality, mobility, sleep quality, general health and quality of life of the population.^1^ Therefore, studies on multisite pain are important. In addition, high public costs are generated through treatment of symptoms and the drop in productivity due to multisite pain.

The present study may contribute to knowledge of the current condition of musculoskeletal symptoms and to identifying possible associations between the presence of multisite pain and predictor variables, since population-based research studies on this outcome are scarce. It may also assist other studies that have the aim of gaining greater understanding of the causes and thus creating interventions at various levels of healthcare.

## OBJECTIVES

The objectives of the present study were to ascertain the prevalence of multisite pain in the last 12 months and in the last seven days, in a population-based sample of adults aged 20 years or over residing in the city of Bauru (São Paulo, Brazil) and to investigate the association of this multisite pain with demographic, socioeconomic, behavioral, reported morbidity and ergonomic variables.

## METHODS

### Study design and ethics

A cross-sectional study was carried out in the urban area of the city of Bauru, which is located in the center-west of the state of São Paulo, Brazil, with approximately 337,094 inhabitants, of whom 207,021 inhabitants are over 20 years of age. This project was approved by the Ethics Committee for Research on Human Beings, of Universidade do Sagrado Coração, Bauru, São Paulo, Brazil, under document no. 201/11, dated August 21, 2018.

### Participants

This study was based on data that were collected through the project “Musculoskeletal symptoms, autonomy and quality of life in the population of Bauru, São Paulo”, which was funded by Fundação de Amparo à Pesquisa do Estado de São Paulo (FAPESP), through procedural number 2011/20123-4, and by Conselho Nacional de Desenvolvimento Científico e Tecnológico (CNPq), through procedural number 478188/2011-0.

Age and sex groups (called sample domains) were firstly defined with a minimum number of individuals per sample, in order to allow further analysis. Six sample domains were thus determined: 20 to 35-year-old men; 20 to 35-year-old women; 36 to 59-year-old men; 36 to 59-year-old women; 60-year-old and older men; and 60-year-old and older women.^9,10^

The sample size calculation was based on the following premises: an estimated proportion of 50% of the population subgroups, since this is the maximum variability that leads to obtaining conservative sample sizes; a 95% confidence level for estimation of confidence intervals; a 10% sampling error, indicating that the amplitude between the estimated sample and the population parameter should not exceed this value; and a design effect of 2. Through this calculation, the sample size for each age group (20 to 35 years, 36 to 59 years and 60 years or over) was determined to be a minimum of 200 individuals (100 males and 100 females). Thus, the total sample size was determined to be 600 participants living in the city of Bauru, SP, Brazil.^9,10^

The sample was randomly selected using a two-stage cluster design. The sampling units were obtained from the 2011 National Household Sample Survey, which provided a list of the private addresses in each census tract. Fifty urban census tracts were randomly selected from the 476 tracts identified. These census tracts constituted the primary sampling units (PSUs). The households were the secondary sampling units (SSUs). The PSUs were randomly selected by means of systematic sampling with a probability proportional to their sizes.^9,10^

For each census tract, the number of households to be randomly selected was determined according to the ratio of the average number of individuals per household in each sample domain.^9^ It was estimated, therefore, that about 12 households per census tract should be visited. These households were randomly selected using a system that took into consideration all eligible individuals residing in the households. In households with more than one eligible subject, all subjects within the age range of each group were considered eligible for the interviews. In the event of refusal of one or all subjects, a new household was randomly selected.

Individuals who could not be located even after four visits were considered to be losses. In these cases, at least one visit was attempted in the evening and one on the weekend. Individuals who were unable to respond because of travel or who refused to answer the questionnaire through their personal choice were also considered to be losses.^9,10^

### Data collection procedures

The interviews were conducted by trained interviewers and the fieldwork was supervised by the researchers involved in the study. Data collection took place between February and June 2012. The coding was performed by the interviewers and reviewed by the chief researcher. The supervisors also performed quality control, which consisted of applying a short version of the questionnaires to 10% of the respondents.^9,10^

Interviews were conducted with all residents aged twenty or over who were living in the selected households in the urban area of Bauru, excluding people with mental and physical disabilities. Older participants were asked to do the Mini-Mental State Examination at the beginning of the interview and those who scored below 27 were considered as having cognitive loss and therefore were excluded from the study.^9,10^

### Instruments

The Nordic Pain Questionnaire, validated and adapted to Brazilian culture,^12^ was used to collect data on pain in specific body regions (neck, shoulders, thoracic spine, elbows, wrists/hands, lumbar spine, hips/thighs, knees and ankles/feet). This questionnaire presents a drawing of the human body with the names of its different regions highlighted, so that interviewees can better specify the region where their pain is. At the time of the interview, each subject answered the following questions: 1. have you felt any pain in this body region (e.g. shoulders) in the last twelve months? 2. have you felt any pain in this body region (e.g. shoulders) in the last seven days? This procedure was repeated for all regions, and all questions allowed only a yes or no answer. From these data, we created the variable “multisite pain”, which was defined as pain in two or more sites (≥ 2 sites) in the last 12 months and in the last seven days.^1–3^

To characterize the subjects, the following data were collected, as previously reported.^9,10^ The individual factors included sex, age (20-35, 36-59 or ≥ 60 years), body mass index (< 18.5; ≥ 18.5 and < 25; or ≥ 25 kg/m^2^), marital status (single, married or widowed/separated), education (0-4, 5-8, 9-11 or 12 years or over), skin color or race (white, black, brown, indigenous or East Asian),^13^ income (low: up to 3 times the minimum monthly wage (MW); medium: from 4 to 9 MW; or high: 10 or more MW).

The participants were asked the following questions regarding their use of electronic devices (time spent watching TV and on the computer and/or playing video games):^9,10^ “Do you watch TV in a normal week?” (yes or no); “How many times do you watch TV in a normal week?” (up to two times; three to four times; or five times or more in the week); “How many hours do you watch TV on a normal day?” (up to 2 hours or 3 hours or more per day); “How often do you use a computer or play video games in a normal week?” (up to two times; three to four times; or five times or more per week); “For how many hours do you use a computer or play video games on a normal day?” (up to 2 hours or 3 hours or more per day).

The ergonomic variables were characterized in terms of the interviewee’s perception of the frequency of exposure, which was identified from among the options of never, rarely, usually or always. The variables measured were physical effort, vibration and repetitiveness, and also any occurrence of an incorrect position, characterized by the frequencies with which the interviewee worked/studied in a sitting, standing, squatting, lying down or kneeling position. In the case of retired and unemployed individuals, they were asked to answer considering the activities that they habitually performed. In order to define the association between multisite pain and ergonomic variables, the frequencies obtained in the categories “never” and “rarely” were added together and categorized into a single group; the same was done for the categories “generally” and “always”.^10,14^

Individuals who reported smoking every day (at least one cigarette per day) or occasionally (less than one cigarette per day) were considered to be smokers; and individuals who had stopped smoking at least six months before the interview were considered to be former smokers.^10,14^

Information on morbidity was collected through the interview, in which the subjects answered the question: “among the alternatives below (hypertension, osteoporosis, diabetes, osteoarthritis, respiratory, gastrointestinal diseases and urinary system diseases), choose the one/ones that matches/match a diagnosis that you received from a doctor in the last 12 months”.^9^

The International Physical Activity Questionnaire (IPAQ), as validated for the Brazilian population, was used to check each subject’s physical activity level. A threshold of 150 minutes of physical activity per week was established for classifying individuals as active (150 minutes per week or more) or insufficiently active (below 150 min per week).^15^

### Statistical analysis

The Statistical Package for the Social Sciences (SPSS), version 18.0 (IBM Corp., Armonk, New York, United States) was used to analyze the data. The data were entered by an undergraduate student who did not participate in the study. 10% of the questionnaires were randomly chosen to test the accuracy of the data typing, and an error was found and corrected. Another 5% were then randomly chosen and no error was found.

The prevalence, confidence intervals and bivariate and Poisson regression analyses between multisite pain and all independent variables were calculated, to determine the significance level and estimated relative risk of the 95% confidence intervals.

Poisson regression analysis with robust variance was performed in accordance with a theoretical-conceptual hierarchical model. A reference category was established, for all variables, which was considered to present the lowest risk. The variables were organized into four levels according to their temporal and causal relationships to multisite pain. Adjustments to the first level were performed using all the variables that belonged to this level. Adjustments to the second level were performed using variables from the previous level that presented P-values < 0.10 and also the variables that belonged to the second level. Adjustments to the third level were performed using variables from the first and second levels with P-values < 0.10, and also the variables that belonged to the third level. The fourth level was controlled for the previous three levels. A regressive selection process was used to determine the variables that would remain in the regression model, such that all the variables with P-values < 0.05 were kept in the model.^16,17^

## RESULTS

A total of 641 individuals from eligible households were considered for inclusion, and 600 of them were actually interviewed. There were 21 losses, for which the main reasons were: “participant absent from home” and “participant set a time with the interviewer but did not show”. There were also 20 refusals, for the following reasons: “participant did not respond to interview request” (n = 12) and “mental disability” (n = 8).

Among the interviewees, most had had between 9 and 11 years of formal education, were of white race, had married marital status, had low income, were non-smokers, were insufficiently active (physical activity level) and reported having diseases (hypertension, diabetes mellitus or depression).

**Table 1** shows that women presented higher prevalence, independent of the sites, both in the last 12 months and in the last seven days. Low back pain was the most prevalent complaint among both men and women in both the past 12 months and the past seven days. Regarding the number of regions with pain, presentation of 1, 2 and up to 3 sites was more prevalent.

**Table 1.** Prevalence of musculoskeletal pain at different sites, according to sex and time period

Musculoskeletal pain				
Parts of the body	Female		Male	
	12 months	7 days	12 months	7 days
	n (%)	n (%)	n (%)	n (%)
Neck	68 (22.7)	31 (10.3)	54 (18.0)	28 (9.3)
Shoulder	89 (29.7)	53 (17.7)	55 (18.6)	29 (9.7)
Thoracic back	84 (28.0)	51 (17.0)	51 (17.0)	34 (11.3)
Elbow	28 (9.3)	12 (4.0)	24 (8.0)	9 (3.0)
Wrist/hands	84 (28.0)	48 (16.0)	34 (11.3)	18 (6.0)
Low back	125 (41.7)	81 (27.0)	80 (26.7)	53 (17.7)
Hip/thighs	69 (23.0)	41 (13.7)	34 (11.3)	22 (7.3)
Knee	78 (26.0)	49 (16.3)	76 (25.3)	46 (15.3)
Ankle/foot	54 (18.0)	32 (10.7)	40 (13.3)	17 (5.7)
**Number of pain sites**				
None	75 (25.0)	138 (46.0)	106 (35.3)	181 (60.3)
1	61 (20.3)	63 (21.0)	79 (26.3)	61 (20.3)
2	56 (18.7)	40 (13.3)	51 (17.0)	30 (10.0)
3	38 (12.6)	27 (9.0)	33 (11.0)	12 (4.0)
4	28 (9.3)	16 (5.3)	15 (5.0)	4 (1.3)
5	13 (4.3)	4 (1.3)	3 (1.0)	1 (0.3)
6	11 (3.6)	3 (1.0)	5 (1.6)	5 (1.6)
7	3 (1.0)	4 (1.3)	3 (1.0)	2 (0.6)
8	7 (2.3)	1 (0.3)	3 (1.0)	2 (0.6)
9	8 (2.6)	4 (1.3)	2 (0.6)	2 (0.6)

The prevalence of multisite pain in the last 12 months was 46.5% (confidence interval, CI 42.5 to 50.5), i.e. 38.3% (CI 33.0 to 43.9) in men and 54.6% (CI 49.0 to 60.2) in women, with a statistically significant difference (P < 0.0001). In the last seven days, the prevalence was 26.1% (CI 22.8 to 29.8), i.e. 19.3% (CI 15.2 to 24.1) in men and 33.0% (CI 27.9 to 38.5) in women, with a statistically significant difference (P < 0.0001).

Among the demographic variables, only female sex was associated with multisite pain in the last 12 months. On the other hand, multisite pain in the last seven days was associated with female sex, individuals with low income and individuals aged 60 years or over (**Table 2**).

**Table 2.** Multivariate analysis on multisite pain and demographic and socioeconomic characteristics

Variables	Multisite pain							
	In last 12 months				In last 7 days			
	Total	n	%	PR (95% CI)	Total	n	%	PR (95% CI)
**Sex**								
Male	300	136	45.3	1.00	300	201	67.0	1.00
Female	300	164	54.7	1.59 (1.10-2.30)	300	99	33.0	1.84 (1.21-2.80)
**Age groups**								
20-35 years	200	71	25.4	1.00	200	34	21.7	1.00
36-59 years	200	100	35.8	1.49 (0.87-2.54)	200	45	28.7	1.32 (0.75-2.30)
60 or over	200	108	38.7	1.32 (0.70-2.48)	200	78	49.7	2.13 (1.21-3.75)
**Schooling (years)**								
12 or higher education	105	45	16.1	1.00	105	21	13.4	1.00
9-11	244	105	37.6	0.54 (0.24-1.23)	244	53	33.8	0.50 (0.21-1.19)
5-8	129	64	22.9	0.82 (0.40-1.67)	129	37	23.6	0.99 (0.45-2.18)
0-4	122	65	23.3	0.87 (0.49-1.52)	122	46	29.3	0.78 (0.40-1.53)
**Race**								
White	480	218	78.1	1.00	480	119	75.8	1.00
Black	38	24	8.6	1.52 (0.68-3.37)	38	17	10.8	2.00 (0.96-4.45)
Mixed	82	37	13.3	1.13 (0.64-1.99)	82	21	13.8	1.12 (0.60-2.10)
**Marital status**								
Married	345	147	52.7	1.00	345	85	54.1	1.00
Single	150	66	23.7	1.29 (0.81-2.05)	150	31	19.7	1.32 (0.70-2.49)
Widowed/divorced	105	66	23.7	1.40 (0.83-2.35)	105	41	26.1	1.03 (0.57-1.86)
**Income**								
High	71	30	10.8	1.00	71	9	5.7	1.00
Medium	140	58	20.8	1.52 (0.82-2.80)	140	31	19.7	2.01 (0.88-4.61)
Low	389	191	68.5	1.25 (0.64-2.47)	389	117	74.5	2.95 (1.34-6.26)

PR = prevalence ratio; CI = confidence interval.

It was noticed that smoking was a protective factor for individuals who reported being former smokers. Individuals who reported having hypertension, diabetes mellitus or depression presented an association with pain in the last 12 months and in the last seven days (**Table 3**).

**Table 3.** Multivariate analysis on multisite pain in relation to physical activity levels, reported diseases and smoking

Variables	Multisite pain							
	In last 12 months				In last 7 days			
	Total	n	%	PR (95% CI)	Total	n	%	PR (95% CI)
**Smoking**								
Nonsmokers	363	167	59.9	1.00	363	91	58.0	1.00
Former smoker	128	53	19.0	0.61 (0.38-0.99)	128	34	21.7	0.85 (0.48-1.50)
Smoker	109	59	21.1	1.20 (0.78-2.10)	109	32	20.4	1.33 (0.75-2.39)
**Hypertension**								
No	402	153	54.8	1.00	402	76	48.4	1.00
Yes	198	126	45.2	2.26 (1.53-3.33)	198	81	51.6	2.23 (1.47-3.38)
**Diabetes mellitus**								
No	531	232	82.3	1.00	531	124	79.0	1.00
Yes	69	47	16.8	2.75 (1.61-4.69)	69	33	21.0	1.79 (1.02-3.17)
**Depression**								
No	520	224	80.3	1.00	520	120	76.4	1.00
Yes	80	55	19.7	2.01 (1.17-3.45)	80	37	23.6	2.02 (1.20-3.42)
**Gastrointestinal disease**								
No	545	238	85.3	1.00	545	132	90.8	1.00
Yes	55	41	14.7	1.79 (0.93-3.33)	55	25	9.2	2.06 (0.97-3.72)
**Renal disease**								
No	568	255	91.4	1.00	568	141	94.7	1.00
Yes	32	24	8.6	2.11 (0.87-5.13)	32	16	5.3	1.79 (0.80-3.99)
**Respiratory disease**								
No	554	251	90.0	1.00	554	140	89.2	1.00
Yes	46	28	10.0	1.61 (0.84-3.10)	46	17	10.8	1.44 (0.73- 2.82)
**Physical activity level**								
Active	210	97	34,8	1.00	210	52	33.1	1
Sedentary	390	182	65.2	0.92 (0.62-1.38)	390	105	66.9	0.99 (0.62-1.57)

PR = prevalence ratio; CI = confidence interval.

Multisite pain in the last 12 months was associated with the variable of watching TV more than three times a week (**Table 4**).

**Table 4.** Multivariate analysis on multisite pain according to use of electronic devices

Variables	Multisite pain							
	In last 12 months				In last 7 days			
	Total	n	%	PR (95% CI)	Total	n	%	PR (95% CI)
**Watching TV**								
No	34	17	6.1	1.00	34	7	4.5	1.00
Yes	566	262	93.9	0.33 (0.91-1.23)	566	150	95.5	0.79 (0.16-3.80)
**Number of times watching TV per week**								
Up to 2	26	5	1.8	1.00	26	4	2.5	1.00
3 or over	540	257	47.6	3.56 (1.30-9.72)	540	146	27.0	1.50 (0.50-4.54)
**Number of hours of TV per day**								
Up to 2	303	137	49.1	1.00	303	75	47.8	1.00
3 or over	263	125	44.8	1.05 (0.70-1.58)	263	75	47.8	1.15 (0.74-1.80)
**Use of computer/videogame**								
No	314	162	58.1	1.00	314	106	67.5	1.00
Yes	286	117	41.9	1.42 (0.90-2.24)	286	51	32.5	0.20 (0.01-3.64)
Number of times using computer/videogame per week								
Up to 2	37	20	7.2	1.00	37	4	2.5	1.00
3 or over	249	98	39.4	0.51 (0.25-1.05)	249	48	19.3	1.97 (0.66-5.82)
**Number of hours of computer/videogame per day**								
Up to 2	159	76	27.2	1	159	31	19.7	1.00
3 or over	127	42	15.1	0.71 (0.44-1.14)	127	21	13.4	0.84 (0.40-1.79)

PR = prevalence ratio; CI = confidence interval.

Multisite pain was also significantly associated with use of a seated position in the last 12 months and in the last seven days (**Table 5**).

**Table 5.** Multivariate analysis on multisite pain according to work-related variables

Variables	Multisite pain							
	In last 12 months				In last 7 days			
	Total	n	%	PR (95% CI)	Total	n	%	PR (95% CI)
**Repetitive movements**								
Never/rarely	240	104	37.3	1.00	240	55	35.0	1.00
Always/usually	360	175	62.7	1.00 (0.66-1.51)	360	102	65.0	1.49 (0.95-2.32)
**Vibration/shaking**								
Never/rarely	505	230	82.4	1.00	505	127	80.9	1.00
Always/usually	95	49	17.6	1.09 (0.60-1.98)	95	30	19.1	1.45 (0.79-2.65)
**Loading and transporting weights**								
Never/rarely	400	177	63.4	1.00	400	106	67.5	1.00
Always/usually	200	102	36.6	1.51 (0.99-2.30)	200	51	32.5	0.77 (0.45-1.31)
**Kneeling**								
Never/rarely	521	240	86.0	1.00	521	134	85.4	1.00
Always/usually	79	39	14.0	1.32 (0.72-2.40)	79	23	14.6	1.32 (0.67-2.59)
**Lying down position**								
Never/rarely	570	267	95.7	1.00	570	148	94.3	1.00
Always/usually	30	12	4.3	0.74 (0.30-1.82)	30	9	5.7	1.00 (0.35-2.85)
**Seated position**								
Never/rarely	223	100	35.8	1.00	551	134	85.4	1.00
Always/usually	377	179	64.2	1.73 (1.13-2.67)	49	23	14.6	4.10 (2.01-8.36)
**Sitting and lifting loads**								
Never/rarely	551	254	91.0	1.00	223	50	31.8	1.00
Always/usually	49	25	9.0	1.06 (0.48-2.31)	377	107	68.2	1.44 (0.86-2.40)
**Sitting and leaning**								
Never/rarely	444	198	71.0	1.00	444	107	68.2	1.00
Always/usually	156	81	29.0	0.99 (0.60-1.64)	156	50	31.8	1.21 (0.69-2.13)
Standing position								
Never/rarely	142	60	21.5	1.00	142	37	23.6	1.00
Always/usually	458	219	78.5	0.97 (0.59-6.85)	458	120	76.4	1.46 (0.83-2.56)
**Standing and leaning**								
Never/rarely	309	128	45.9	1.00	309	83	52.9	1.00
Always/usually	291	151	54.1	1.53 (0.99-2.35)	291	74	47.1	0.63 (0.41-0.97)

PR = prevalence ratio; CI = confidence interval.

## DISCUSSION

The variables associated with multisite pain in the last 12 months were female sex, presence of hypertension, diabetes mellitus or depression, watching TV more than three times a week and working in a seated position. On the other hand, formerly smoking was a protection factor. The associations in the last seven days were with female sex, age group 60 years or over, low income, presence of comorbidities of hypertension, diabetes mellitus or depression and working in a seated position. Multisite musculoskeletal pain had high prevalence in the population studied, like in other countries.^8,18,19^

Female sex was associated with multisite pain in the last 12 months and in the last seven days, thus corroborating the findings of other studies.^8,20^ The difference between the sexes can be explained by the fact that women report and seek more support for musculoskeletal pain, are more exposed to physical factors, psychosocial factors and stress, have less strength than men and perform a double working day.^21^

In the present study, the age group above 60 years was associated with presence of multisite pain in the last seven days, unlike in studies in Estonia and Norway, where it was associated with multisite pain in the last 12 months.^3,20^ Associations between aging and pain are multidimensional and, with advancing age, pain problems become highly complex due to multiple comorbidities.^22,23^ Although multimorbidity becomes more common with age, more than half of the individuals with multimorbidity and nearly two-thirds of those with comorbidities relating to physical and mental health are under 65 years old.^23,24^

The outcome in the last seven days was associated with low income level., like in the study in Norway,^20,24^ which highlighted that low education level and low income interfere to give rise to chronic pain.^24^ The hypotheses that may explain this finding may include lack of access to healthcare services. In addition, individuals with low socioeconomic status do not take self-care actions, such as healthy lifestyle habits, and they work in occupations in which they are at risk of musculoskeletal injury.^24^

Regarding smoking, being a former smoker was a protective factor for multisite pain, thus contradicting other studies,^6,25^ and this difference may be due to the methods used to evaluate cigarette use. However, a study conducted among former smokers with low back pain showed that they had lower risk of seeking therapeutic services than did current smokers, thus suggesting that the effects of smoking may be at least partially reversible.^6^

Multisite pain in the present investigation was associated with individuals who reported depression, thus corroborating the findings of other studies.^24,26^ It had previously been reported that depression was associated with other factors (insomnia and social participation) and that, over the long term, it would contribute to the onset and increased symptoms of chronic musculoskeletal pain, probably due to central sensitization.^26^

In the present study, multisite pain was associated with hypertension, which corroborated the findings from some studies,^26,27^ while this association was not noticed in other studies.^28,29^ One possible explanation for this difference may have been the phenomenon of hyperalgesia in association with hypertension, caused by an interaction between cardiovascular and pain regulation systems.^28,29^

Multisite pain, in the present investigation, was associated with occurrences of individuals who reported diabetes, which corroborated the results from other studies.^16,17,30^ Previous studies had indicated that pain and diabetes probably had relationships with vascular insufficiency, peripheral neuropathy, osteoporosis, obesity, sedentary lifestyle and other factors.^16^ There is evidence suggesting that people with diabetes usually have other comorbidities (e.g. hypertension and dyslipidemia), thus resulting in a more severe clinical picture and consequently increased signs of musculoskeletal pain.^17^

Watching TV more than three times a week and working in a seated position were always associated with multisite pain in the last 12 months, thus corroborating one other investigation^31^ but diverging from another study.^32^ Moreover, it has been reported that the relationship between pain and a seated position would be due to the fact that this position immobilizes the skeletal structures, thereby increasing the demands of muscles, ligaments and other tissues (tissue stress), especially in unfavorable postures. The effect would be associated with the length of stay in the same position, together with low muscle activation, thus leading to pain.^33^

Carrying out occupational activities in seated positions was found in the present study to be associated with multisite pain in the last 12 months and seven days, similarly to the findings from previous studies conducted in Brazil and Finland.^8,34^ Occupational activities that require physical demands cause individuals to use various body segments to perform this task, thus contributing to multisite musculoskeletal pain, and this has been found to be reported more frequently in the last seven days.^35^

This study has some limitations. We did not collect data on psychosocial factors, the frequency, severity, intensity and duration of multisite pain, or how multisite pain at multiple sites affected and/or limited the subjects’ usual activities. The main contributions and strengths of the study are its use of validated questionnaires for the outcome studied and the large number of individuals interviewed. This study also indicated the factors associated with musculoskeletal pain in multi-site pain in a Brazilian population sample, which is an important contribution, given that in Brazil there is a scarcity of sources of data on multisite pain. Moreover, it has been emphasized in the literature that there is a the need to consider the number of regions with pain, together a need to collect data through interviews in order to reduce the information bias.^3,26^ This study will make a contribution as a reference point for epidemiological investigations with a prospective design that aim to evaluate predictors, causality and clinical evolution, and possibly for systematic reviews and meta-analyses.

## CONCLUSION

High prevalence of multisite pain was observed. It was greater in women, both in the last seven days and in the last 12 months, with a statistically significant difference. The variables associated with multisite pain in the last 12 months were female sex, presence of the comorbidities of hypertension, diabetes mellitus or depression, watching TV more than three times a week and working in a seated position, while formerly smoking was a protection factor. The associations in the last seven days were with female sex, age group 60 years or over, low income, presence of the comorbidities of hypertension, diabetes mellitus or depression and working in a seated position.

## Additional information:

The data and materials are available at: https://zenodo.org/deposit/118724/
